# Lessons learned from the DAPA-HF trial concerning the mechanisms of benefit of SGLT2 inhibitors on heart failure events in the context of other large-scale trials nearing completion

**DOI:** 10.1186/s12933-019-0938-6

**Published:** 2019-10-04

**Authors:** Milton Packer

**Affiliations:** 1 Baylor Heart and Vascular Institute, Baylor University Medical Center, 621N. Hall Street, Dallas, TX 75226 USA; 2 Imperial College, London, UK

**Keywords:** SGLT2 inhibitors, Heart failure, Dapagliflozin

## Abstract

Four large-scale trials in type 2 diabetes have shown that sodium-glucose cotransporter 2 (SGLT2) inhibitors prevent the occurrence of serious heart failure events. Additionally, the DAPA-HF trial demonstrated a benefit of dapagliflozin to reduce major adverse outcomes in patients with established heart failure with a reduced ejection fraction. The trial sheds light on potential mechanisms. In DAPA-HF, the benefits of dapagliflozin on heart failure were seen to a similar extent in both patients with or without diabetes, thus undermining the hypothesis that these drugs mitigate glycemia-related cardiotoxicity. The action of SGLT2 inhibitors to promote ketogenesis is also primarily a feature of the action of these drugs in patients with diabetes, raising doubts that enhanced ketogenesis contributes to the benefit on heart failure. Also, dapagliflozin does not have a meaningful effect to decrease circulating natriuretic peptides, and it did not potentiate the actions of diuretics in DAPA-HF; moreover, intensification of diuretics therapy does not reduce cardiovascular death, questioning a benefit of SGLT2 inhibitors that is mediated by an action on renal sodium excretion. Finally, although hematocrit increases with SGLT2 inhibitors might favorably affect patients with coronary artery disease, in DAPA-HF, the benefit of dapagliflozin was similar in patients with or without an ischemic cardiomyopathy; furthermore, increases in hematocrit do not favorably affect the clinical course of patients with heart failure. Therefore, the results of DAPA-HF do not support many currently-held hypotheses about the mechanism of action of SGLT2 inhibitors in heart failure. Ongoing trials are likely to provide further insights.

Four cardiovascular outcome trials in patients with type 2 diabetes have shown that sodium-glucose cotransporter 2 (SGLT2) inhibitors have a remarkable effect to prevent the occurrence of serious heart failure events, largely in patients who did not have a prior diagnosis of heart failure [1]. The magnitude of the benefit has been consistent across trials, and a similar pattern of response has not been reported in studies of other antihyperglycemic drugs. In most instances, the finding of a benefit of SGLT2 inhibitors on heart failure events was not the primary endpoint of these trials; the phenotype of heart failure was not characterized; and the adequacy of background therapy was not established. Therefore, for many years, physicians remained uncertain whether the benefit on heart failure represented a generalizable finding. However, in a recent trial of dapagliflozin in patients with confirmed and well-treated heart failure (DAPA-HF), SGLT2 inhibition reduced the risk of cardiovascular death and hospitalization for heart failure [2]. The effect was clinically and statistically persuasive.

## What was known before the DAPA-HF trial?

In four large-scale clinical trials involving > 36,000 patients with type 2 diabetes followed for 2–5 years who largely did not have a diagnosis of heart failure at the time of study entry, patients treated with a SGLT2 inhibitor experienced a ≈ 25–35% lower risk of hospitalization for heart failure [1]. These benefits emerged shortly following the initiation of therapy and persisted for the entire duration of double-blind treatment. Three different SGLT2 inhibitors (i.e., empagliflozin, canagliflozin and dapagliflozin) have been shown to prevent the development of heart failure in patients at high cardiovascular risk. Only ≈ 10–15% of the patients in these trials had a diagnosis of heart failure at the time of trial enrollment [1].

Patients with type 2 diabetes are at risk of developing two distinct phenotypes of heart failure. Many develop heart failure with a reduced ejection fraction (HFrEF), a phenotype that is typically characterized by the loss and stretch of cardiomyocytes resulting in marked left ventricular (LV) enlargement and striking increases in circulating natriuretic peptides. These patients show a favorable response to drugs that interfere with neurohormonal systems (inhibitors of the renin-angiotensin system, beta-blockers, mineralocorticoid receptor antagonists and neprilysin inhibitors). Yet, many other patients with type 2 diabetes develop heart failure with a preserved ejection fraction (HFpEF), a phenotype that is characterized by systemic and adipose tissue inflammation, resulting in microvascular dysfunction and fibrosis of the myocardium. These patients do not show striking increases in LV size or levels of natriuretic peptides, and they respond modestly and inconsistently to neurohormonal antagonists that are effective in patients with HFrEF.

Do SGLT2 inhibitors exert preferentially favorable effects on HFrEF or HFpEF? The DECLARE-TIMI 58 trial collected information on LV ejection fraction measured *before* randomization, and it showed that the reduction in the risk of heart failure hospitalizations with dapagliflozin was particularly striking in patients who had HFrEF at the time of enrollment; furthermore, cardiovascular death was reduced only in patients with HFrEF and not in patients with HFpEF [3]. Conversely, in the CANVAS trials, investigators characterized the phenotype of heart failure at the time that heart failure events occurred *after* randomization; interestingly, they also observed the most marked risk reduction in patients who developed HFrEF during the course of follow-up [4].

Many mechanisms have been postulated to explain these benefits of SGLT2 inhibitors. Even though none of the trials could discern any relation between changes in glycemic control and the effects on heart failure, some researchers believed that mitigation of glycemia-related cardiotoxicity could contribute importantly to the observed reduced risk [5]. Other investigators suggested that the natriuretic action of SGLT2 inhibitors could cause a reduction in plasma volume or interstitial fluid, which might reduce cardiac volumes and favorably influence ventricular remodeling [6]. Still others suggested that SGLT2 inhibitors might favorably influence energy balance in the failing heart by increasing circulating levels of ketone bodies and by promoting a shift in myocardial fuel utilization from glucose to ketones and free fatty acids [7]. Additionally, SGLT2 inhibitors were postulated to enhance the synthesis of erythropoietin, with the expectation that an increase in red cell mass might increase oxygen delivery to the myocardium, which could be important in those with underlying ischemic heart disease [8]. Importantly, a benefit of SGLT2 inhibitors to prevent heart failure events could not be explained by an action to reduce the risk of coronary artery occlusive events, since these drugs do not prevent myocardial infarction [9].

## What is now known after the DAPA-HF trial?

The DAPA-HF trial randomly assigned 4744 patients with chronic heart failure to placebo or dapagliflozin who were treated and followed for an average of 18.2 months. The trial enrolled only patients with HFrEF, and it ensured that all patients were receiving appropriate background treatments with neurohormonal antagonists. Specifically, > 90% were receiving inhibitors of the renin-angiotensin system and beta-blockers, and > 70% were receiving mineralocorticoid receptor antagonists. Approximately half of the patients had diabetes at baseline, and > 40% did not have underlying ischemic heart disease.

In the trial, when added to currently accepted treatments for heart failure, dapagliflozin produced a 26% reduction in the combined risk of cardiovascular death, hospitalization for heart failure or an urgent visit for worsening heart failure requiring intravenous therapy (P = 0.00001) [2]. In addition, dapagliflozin reduced cardiovascular death by 18% (P = 0.029) and all-cause mortality by 17% (P = 0.022). Importantly, the magnitude of benefit on the primary composite endpoint was similar in patients with and without diabetes and in patients with or without ischemic heart disease.

The DAPA-trial is the first large-scale trial to demonstrate a convincing effect of SGLT2 inhibitors to reduce major adverse cardiovascular outcomes in patients with established heart failure. The trial extends observations from earlier trials in type 2 diabetes that showed a striking benefit of these drugs in preventing heart failure events in patients who have or develop HFrEF. Perhaps more interestingly, the trial also provides insights into previously proposed mechanisms by which SGLT2 inhibitors might exert their benefits.

*First*, in DAPA-HF, the benefits of dapagliflozin on the progression of heart failure were seen to a similar extent in both patients with or without diabetes, thus undermining the hypothesis that mitigation of glycemia-related cardiotoxicity by SGLT2 inhibitors slows the progression of heart failure [5].

*Second*, the benefits of dapagliflozin in DAPA-HF were seen in patients receiving loop diuretics that were sufficient to keep their mean circulating levels of N-terminal proBNP < 1500 pg/ml. Although SGLT2 inhibition may theoretically potentiate the natriuretic action of prescribed diuretics, their use has not been accompanied by a meaningful decrease in circulating natriuretic peptides [10, 11]. In the DAPA-HF trial, dapagliflozin produced only a 10–15% decline in N-terminal proBNP, an effect size less than that seen with conventional diuretics [12, 13], and use of the drug did not alter the dosing or enhance the effects of prescribed diuretics. Furthermore, intensification of diuretic therapy has typically been associated with an increased risk of cardiovascular and sudden death in patients with heart failure [14]. Yet, in the diabetes cardiovascular outcomes trials and in DAPA-HF, SGLT2 inhibition reduced cardiovascular death as well as sudden death [1]. Consequently, the totality of evidence does not support a benefit of these drugs that is mediated by an action on renal sodium excretion.

*Third*, patients with chronic heart failure have increased circulating levels and utilization of ketone bodies in the absence of treatment with an SGLT2 inhibitor [15, 16]. The infusion of ketone bodies in patients with heart failure and a reduced ejection fraction leads to increases in cardiac contractility and heart rate; [17] yet, such positive inotropic and chronotropic effects are not seen with the use of SGLT2 inhibitors in clinical trials. Most importantly, the action of SGLT2 inhibitors to promote ketogenesis is primarily a feature of the action of these drugs in patients with type 2 diabetes; this ketogenic effect is attenuated in nondiabetic patients [18]. Yet, in DAPA-HF, the magnitude of the benefit of dapagliflozin in reducing serious heart failure events was similar in patients with or without diabetes, raising doubts that enhanced ketogenesis contributed importantly to the benefit on heart failure.

*Fourth*, although hematocrit increases with SGLT2 inhibitors might have favorable effects in patients with coronary artery disease, in DAPA-HF, there was no difference in the magnitude of benefit of dapagliflozin in patients with or without an ischemic cardiomyopathy. Furthermore, increases in hematocrit produced by erythropoietin-mimetic agents in patients with heart failure do not have favorable effects on the course of the disease [19].

## Summary and conclusions

The DAPA-HF trial substantially extends the benefits of SGLT2 inhibitors in slowing the evolution and progression of heart failure, particularly in patients with a reduced ejection fraction. To date, five large-scale trials (that have enrolled patients with and without diabetes) have reported results that are clinically important and remarkably consistent, both qualitatively and quantitatively. Additionally, the findings of the DAPA-HF trial raise serious doubts about the validity of current assumptions about the physiological mechanisms that have been proposed to explain the benefits of SGLT2 inhibitors. Instead, the benefits of SGLT2 inhibitors in heart failure are most likely related to an action of these drugs to promote mechanisms of cardiomyocyte viability and ameliorate pathways that lead to cardiomyocyte death [20].

A second large-scale trial of SGLT2 inhibition in heart failure is nearing completion. The EMPEROR-Reduced trial has enrolled > 3600 patients with HFrEF, with and without type 2 diabetes and with or without ischemic heart disease, who have been randomized to receive treatment with empagliflozin or placebo in addition to all appropriate treatments for heart failure. The EMPEROR-Reduced trial is designed to target the recruitment of patients with heart failure that is more advanced than in those enrolled in DAPA-HF, as reflected by the requirement for lower levels of LV ejection fraction and higher circulating levels of natriuretic peptides. Extensive biomarker studies in the trial are poised to support new hypotheses about the potential mechanisms by which SGLT2 inhibitors exert their benefits on heart failure.

Finally, two trials (the EMPEROR-Preserved trial with empagliflozin and the DELIVER trial with dapagliflozin) are evaluating the effects of SGLT2 inhibitors in > 10,000 patients with an established diagnosis of HFpEF across the two trials. Demonstration of a benefit of SGLT2 inhibitors on heart failure events in these studies would dramatically broaden the clinical impact of these drugs.

## Data Availability

Not applicable.

## Abbreviations

DAPA-HF: Dapagliflozin and Prevention of Adverse Outcomes in Heart Failure
HFpEF: heart failure with preserved ejection fraction
HFrEF: heart failure with reduced ejection fraction
LV: left ventricular
SGLT2: sodium–glucose cotransporter 2
